# Development and Regeneration of Muscle, Tendon, and Myotendinous Junctions in Striated Skeletal Muscle

**DOI:** 10.3390/ijms23063006

**Published:** 2022-03-10

**Authors:** Masahito Yamamoto, Koji Sakiyama, Kei Kitamura, Yutaro Yamamoto, Takahiro Takagi, Sayo Sekiya, Genji Watanabe, Shuichiro Taniguchi, Yudai Ogawa, Satoshi Ishizuka, Yuki Sugiyama, Takeshi Takayama, Katsuhiko Hayashi, Wei-Jen Chang, Shinichi Abe

**Affiliations:** 1 Department of Anatomy, Tokyo Dental College, 2-9-18 Kanda-Misakicho, Chiyoda-ku, Tokyo 101-0061, Japan; yamamotomasahito@tdc.ac.jp (M.Y.); yutaroyamamoto@tdc.ac.jp (Y.Y.); takagitakahiro@tdc.ac.jp (T.T.); sekiyas@tdc.ac.jp (S.S.); watanabegenji@tdc.ac.jp (G.W.); taniguchisyuuichirou@tdc.ac.jp (S.T.); sugiyamayuuki@tdc.ac.jp (Y.S.); 2 Division of Anatomy, Department of Human Development and Fostering, School of Dentistry, Meikai University, 1-1 Keyaki-dai, Sakado-shi 350-0283, Japan; sakiyama@dent.meikai.ac.jp; 3 Department of Histology and Developmental Biology, Tokyo Dental College, 2-9-18 Kanda-Misakicho, Chiyoda-ku, Tokyo 101-0061, Japan; kitamurakei@tdc.ac.jp (K.K.); ogawayudai@tdc.ac.jp (Y.O.); 4 Department of Pharmacology, Tokyo Dental College, 2-9-18 Kandamisaki-cho, Chiyoda-ku, Tokyo 101-0061, Japan; ishidukasatoshi@tdc.ac.jp; 5 Department of Dentistry, The Jikei University School of Medicine, 3-25-8 Nishi-Shimbashi, Minato-ku, Tokyo 105-8461, Japan; tt1110@jikei.ac.jp (T.T.); katsuh@jikei.ac.jp (K.H.); 6 School of Dentistry, College of Oral Medicine, Taipei Medical University, 250 Wu-Hsing Street, Taipei 110, Taiwan; cweijen1@tmu.edu.tw

**Keywords:** HMGB1 protein, small cytoplasmic RNA, staphylococcal protein A, single-cell analysis, skeletal muscle, myoblasts

## Abstract

Owing to a rapid increase in aging population in recent years, the deterioration of motor function in older adults has become an important social problem, and several studies have aimed to investigate the mechanisms underlying muscle function decline. Furthermore, structural maintenance of the muscle–tendon–bone complexes in the muscle attachment sites is important for motor function, particularly for joints; however, the development and regeneration of these complexes have not been studied thoroughly and require further elucidation. Recent studies have provided insights into the roles of mesenchymal progenitors in the development and regeneration of muscles and myotendinous junctions. In particular, studies on muscles and myotendinous junctions have—through the use of the recently developed scRNA-seq—reported the presence of syncytia, thereby suggesting that fibroblasts may be transformed into myoblasts in a BMP-dependent manner. In addition, the high mobility group box 1—a DNA-binding protein found in nuclei—is reportedly involved in muscle regeneration. Furthermore, studies have identified several factors required for the formation of locomotor apparatuses, e.g., tenomodulin (Tnmd) and mohawk (Mkx), which are essential for tendon maturation.

## 1. Introduction

Skeletal muscle fibers are multinucleated myocytes that are formed after several stages; one such stage is myoblast fusion, which occurs as the body grows. Muscle differentiation concurrently occurs during myoblast fusion. Moreover, muscle differentiation occurs intermittently in the musculature of a mature body to adapt to functional changes. Therefore, compared with other tissues in the body, muscle tissues are considered to have excellent plasticity allowing for constant functional and structural changes. Many recent studies on muscles have focused on the relationships of muscles with surrounding tissues and cells, as well as on the muscles themselves. In particular, studies have focused on the relationships of muscles with mesenchymal progenitors in the muscle interstitium and myotendinous junctions.

**Figure 1:**
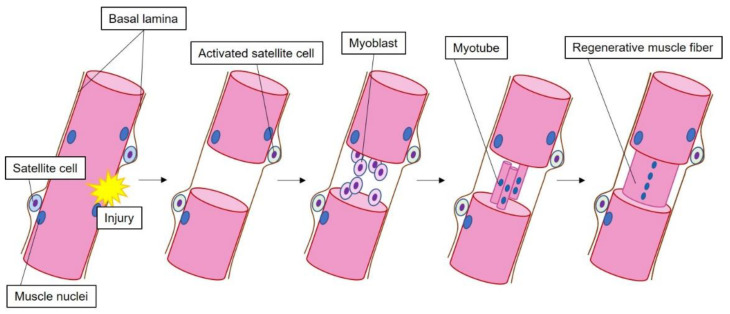
General healing course of muscle fibers.

**Figure 2:**
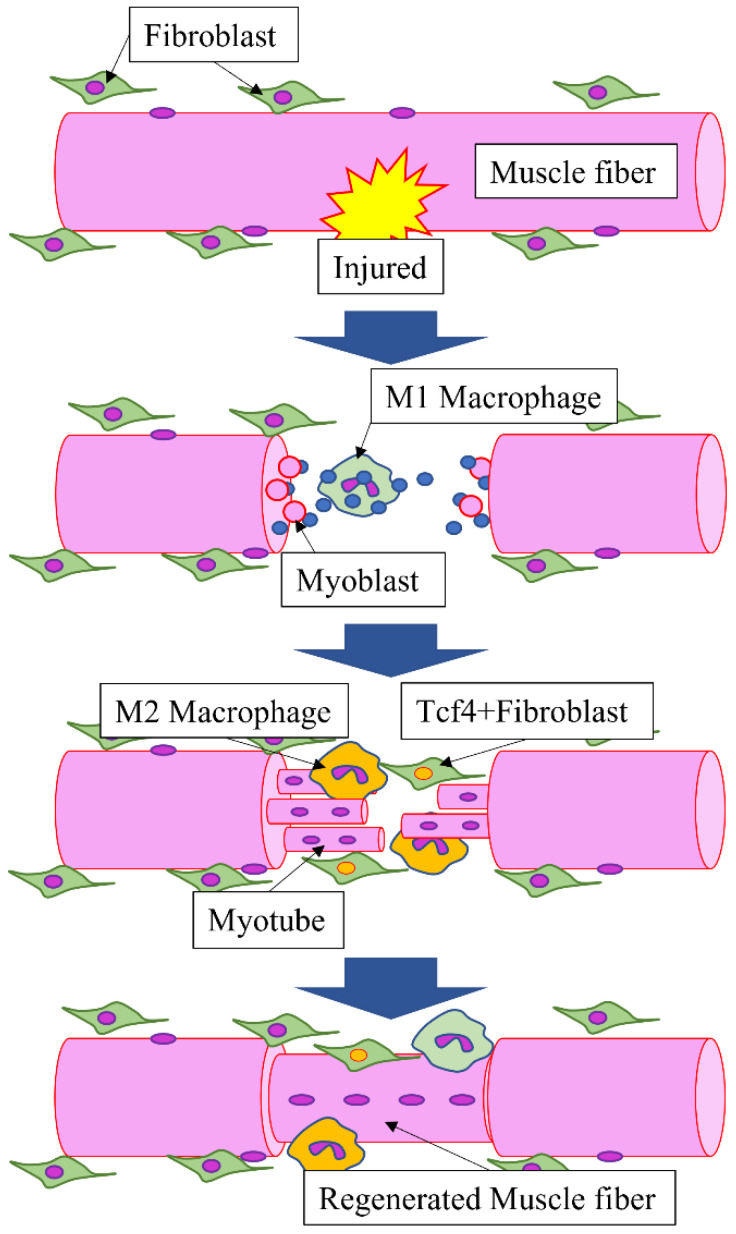
“Tcf4-positive fibroblasts” involved in muscle regeneration.

Uezumi et al. [1] identified mesenchymal progenitor cells which specifically express platelet-derived growth factor receptor alpha (PDGFRα) in the interstitium of muscle tissue. Furthermore, they have demonstrated that these cells are the point of origin for adipogenesis and fibrosis of skeletal muscles. Joe et al. [2] have also shown that adipogenesis and fibrosis originate from cells characterized by CD31 (−), CD45 (−), stem cell antigen-1 (Sca-1) (+), and CD34 (+), and consequently named the cells fibro/adipogenic progenitors (FAPs). Lees-Shepard JB and Eisner C [3,4] subsequently reported that these cells contribute to ectopic bone formation in skeletal muscles. Furthermore, in their study, Neto et al. [5] have demonstrated that niches of telocytes, which have garnered attention as mesenchymal progenitor cells, are found in myotendinous junctions. Telocytes reportedly express mesenchymal stem cell markers, such as PDGFRα, CD34, and Sca-1 [6], which suggests that these may be identical cells. Considering that transcription factor 4 (Tcf4) was reported as a marker of fibroblasts in the muscle interstitium by Mathew et al. [7], the role played by intramuscular fibroblasts in muscle regeneration and development has also been studied. Using Pax7CreERT and Tcf4CreERT mice, Murphy et al. [8] have revealed the involvement of fibroblasts in normal muscle regeneration. Although the relationship between these fibroblasts and non-myogenic mesenchymal progenitors has not been clarified, attention has been drawn to the possibility that they may be identical cells owing to the fact that Tcf4+ cells express PDGFRα [9]. We also focused on these cells and have reported that Tcf4-positive fibroblasts enter the cytoplasm of regenerating muscle during muscle regeneration [10] (Figure 1 and Figure 2).

Through these studies, our knowledge about the role played by mesenchymal progenitors in the development and regeneration of muscle and myotendinous junctions has increased. In this review, we summarize findings on muscle development and regeneration reported thus far.

When muscle damage occurs, muscle satellite cells accumulate at the muscle damage site. Furthermore, muscle satellite cells differentiate. That is, muscle satellite cells become myoblasts and form myotubes. The myotube then becomes a regenerative muscle with a central nuclear.

Tcf4-positive fibroblasts enter the cytoplasm of regenerating muscle during muscle regeneration.

## 2. Part 1: Maturation

### 2.1. Muscle and Myotendinous Junctions

(1) Developmental mechanisms of muscles and myotendinous junctions revealed by scRNA-seq

Recently developed single-nucleus RNA sequencing (scRNA-seq) has been used to obtain some important insights into syncytial muscles and myotendinous junctions [11]. A scRNA-seq analysis of muscle fibers performed by Dos Santos et al. and Petrany et al. [12] has revealed several nuclear subtypes found in muscles, some subtypes of which are previously unknown. Petrany et al. discovered two different myonuclear types at the muscle ends and named them myotendinous junction (MTJ)-A and MTJ-B. The MTJ-A nuclei expressed genes known to be expressed specifically in MTJ, such as Col22a1 and Itgb1, whereas the MTJ-B nuclei expressed a series of collagens known to deposit in MTJs, such as Col1a2, Col6a1, and Col6a3, and genes mainly expressed in the connective tissue, such as Pdgfrb. Unlike the MTJ-A type, the MTJ-B-type nuclei are not necessarily found in all muscle fibers, and express both muscle fiber and connective tissue markers. The results of further analysis focusing on this myonuclear subtype suggests that the MTJ-B subtype represents a population of muscle fiber nuclei that co-express specific genes of muscle fibers and connective tissues (Pdgfrb, Col6a3, and Ebf1).

A myotendinous junction is a tissue located at a junction of myoblasts derived from somites and tenoblasts derived from the lateral plate mesoderm (LPM), and its origin remains largely unclear. Through scRNA-seq analysis of the muscle of newborn mice, Yaseen et al. [13] have reported a “dual identity” nuclear population expressing both muscle fiber and fibrous connective tissue markers. Furthermore, they focused on paired related homeobox 1 (Prrx1), a specific marker of LPM-derived cells, and crossed Prrx1–Cre mice and various fluorescent reporter transgenic mice to attempt cell lineage analysis of the LPM. They showed that LPM-derived fibroblasts expressed myoblast determination protein 1 (MyoD1), a myoblast marker, which demonstrates that fibroblasts are switched to myoblasts and acquire dual identity before fibroblast fusion to myotubes. In addition, these LPM-derived cell nuclei appeared to express lysyl oxidase homolog 3 (LoxL3) and some other factors which play important roles in the MTJ formation, even after a period of muscular fusion. They have stated that these “dual identity” cellular nuclei may be relevant to the MTJ-B myonuclear subtype because they express fibroblast markers such as Pdgfra.

Esteves de Lima et al. [14] conducted a cell lineage analysis experiment transplanting the undifferentiated quail mesoderm into chickens, and found paired box protein Pax-7 (Pax7)-positive LPM-derived cells, i.e., myoblasts. Moreover, lineage analysis of myotome-derived cells by crossing Pax3–Cre mice and Tomato reporter mice led to the discovery of non-myotome-derived Pax7-positive cells at the muscle ends. Furthermore, cell lineage analyses focusing on Scleraxis (CSX), a widely known tendon marker during development and tendon formation, and protein odd-skipped-related 1 (OSR1), a key regulator of muscle connective tissue differentiation, identified Pax7-positive cells originating from CSX and OSR1 lineages, respectively, at the muscle ends. These results have shown the presence of limb connective tissue-derived, non-somite-derived, myogenic cells at the muscle ends. Next, they conducted a scRNA-seq analysis using E4, 6, and 10 muscle fibers. The results identified a population of cells expressing both connective tissue markers and muscle markers (CT/M cells), and suggested that these cells were in the process of transitioning from connective tissue cells to muscle cells. They then explored the signaling pathways involved in the acquisition of the “dual identity” by CT/M cells, with a focus on ID genes and pSMAD1/5/9, which are known as activity markers of bone morphogenetic protein (BMP). The results suggest the presence of BMP-responsive CT/M cells at the muscle–tendon interface. Finally, they conducted an experiment in which a retrovirus was used to overexpress BMP4 or the BMP antagonist NOGGIN. They found that BMP overexpression induced the transformation of fibroblasts into myoblasts, whereas BMP suppression induced transformation into fibroblasts. These findings suggest the possibility that fibroblasts transform into myoblasts in a BMP-dependent manner in the process of limb formation.

(2) Morphogenesis of the tongue dependent on Dlx5 and Dlx6 expression in mice

Research on muscle development has great implications when considering muscle regeneration. Here, we will explain new findings regarding recent developments using the muscles of the tongue as an example.

Tongue development starts in the early embryonic period, and the lingual primordium is formed from two lateral lingual swellings in the first pharyngeal arch, and a median lingual swelling in the third and fourth pharyngeal arches. Then, the three swellings originating from different pharyngeal arches expand and are fused to form the tongue [15,16]. This expansion is due to vigorous cell proliferation of cranial neural crest (CNC)-derived cells inside the mesenchyme and results in the formation of connective tissues, such as the lingual septum. Meanwhile, myoblasts forming lingual muscle are derived from the paraxial mesoderm and migrate anteriorly from the occipital somite, before they enter the lingual mesenchymels, express myogenin and myoD, and differentiate into myoblasts [17,18]. Heude et al. have reported the dependence of this cell−cell interaction on the Dlx5 and Dlx6 expression in CNC cells, and have shown that the lingual muscles are severely reduced and deficient in Dlx5/6^−/−^ mice [19]. Moreover, Wnt5a, a Wnt family member, has been suggested to be involved in unique muscle fiber running patterns of intrinsic and extrinsic muscles of the tongue. Wnt5a binds to its receptor Ror2 to activate CaMKII, and determines differentiation from stem cells to muscle [20]. In Wnt5a^−/−^ mice, the tongue tends to be shorter, and the development of lingual papillae is controlled [21]. Furthermore, the differentiation of myoblasts is regulated, and the orientation regularity of fibers is lost [22]. These findings indicate that the normal development of the quantity and polarity of muscle fibers constituting the tongue greatly contributes to breastfeeding, swallowing, and mastication in the early stages of life.

### 2.2. Tendon

Tendons in the head, trunk, and limbs are derived from the cranial neural crest, sclerotome, and lateral plate mesoderm, respectively. In the 20th century, virtually no key proteins in tendon development were known, and tenascin, which is also expressed in cartilages and nerves, was considered to be a tendon marker [23,24,25]. However, research on tendon development has accelerated considerably in the 21st century, after tenomodulin (Tnmd) and scleraxis (Scx), i.e., an angiogenesis inhibitor and a transcription factor, respectively, were found to be expressed during tendon development [26,27]. Brent et al. (2005) [28] have shown that the expression of scleraxis (Scx) in undifferentiated cells in an early developmental stage destined the cells to become tendons, while Shukunami et al. (2006) [29] and Ito et al. (2010) [30] have shown that Tnmd and mohawk (Mkx<9>) are essential for tendon maturation. In Scx-deficient mice, expression levels of Tnmd, Col14a1, decorin (Dcn), Mkx, and early growth response 1 (Egr1) in the tendons are reduced; therefore, Scx is considered to be the most upstream when compared with the currently known tendon marker genes [31]. Furthermore, Sox9, a transcription factor essential for cartilage development, has been known to be important for tendon development and is expressed in the early and middle stages of tendon development [32].

Little is known currently about the cellular and molecular mechanisms that promote tendon differentiation and maturation in tendon regeneration. The major transcription factors and signaling pathways involved in tendons have originally been discovered through studies on embryonic development [33,34,35,36]. Representative transcription factors reported to date include Scx, Mkx, and Egr1, and the presence of TGF-β and FGF (fibroblast growth factor) is mainly required for the expression of these transcription factors. Among these, Scleraxis (Scx) is still the marker that is known to be expressed in tendon progenitor cells at the earliest stage, and Mohawk (Mkx) is an important regulator of postnatal maturation of collagens [27,30,37,38]. There has been significant progress in our understanding of tendon development, as summarized above. However, we know practically nothing about the biological events organizing tendon differentiation, maturation, and healing-associated scar formation.

## 3. Part 2: Regeneration

### 3.1. Role of HMGB1 during Muscle Regeneration

High mobility group box 1 (HMGB1) is a DNA-binding protein found in cellular nuclei [39,40,41]. However, HMGB1 is released extracellularly when normal cells are destroyed by necrotic or tumor cells, and their nuclear envelope is no longer intact [4]. The released HMGB1 serves as an extracellular signaling molecule through its binding to advanced glycation end products (AGE) [42]. Therefore, the HMGB1 expression has been reported in various diseases, such as malignant tumors, sepsis, and arthritis [42,43,44]. Experiments to map the distribution of HMGB1 expressed in muscle fibers located in the vicinity of tongue cancer lesions confirmed the robust HMGB1 expression around the cancer lesion as a result of extracellular release of HMGB1; this is attributable to the necrosis of the muscle fibers around the cancer lesion [41,45]. Furthermore, recent studies have reported that HMGB1 contributes to the regeneration of damaged tissue [46,47,48,49,50]. As regenerated muscles emerge from these spaces, HMGB1 in normal cells functions to maintain DNA. Once it is released from nuclei after cell necrosis, it contributes to the creation of an environment that helps induce invasion by immune cells and phagocytosis of necrotic cells, and facilitates muscle regeneration.

These findings suggest that HMGB1 is deeply involved in the life and death of cells because HMGB1 affects cellular DNA maintenance and damage and regeneration after necrosis.

### 3.2. Tendon Regeneration in Myotendinous Junctions

Regarding tendon regeneration, recent findings are summarized from the experimental results that also observed myotendinous junctions in mice.

Scx-positive cells (blue) and αSMA-positive cells (red) were accumulated in the stumps of the torn tendon 5 days after surgery; however, only αSMA-positive cells (red) were accumulated, and no Scx-positive cells were found in the defect region (green).

(1) Tendons in neonates and regeneration

A study has demonstrated differences in the regeneration process between tendons in neonates (neonatal tendons; neotendons) and tendons in adult animals [51]. In adult animals, the regeneration process in tendons that underwent complete division (tenotomy) has been known to differ from that in tendons that underwent partial division (transection) [52]; however, the processes largely remain unclear, as mentioned above.

In the regeneration of tendons in neonates (neonatal tendons; neotendons), tendon stem/progenitor cells have been proposed to be present in the epitenon [53,54,55,56,57]. Scx-positive and non-Scx-positive cells have been reported to coexist during the regeneration of tendons after complete division (tenotomy). In neotendons, Scx-positive cells are already found all over the place on day 5 after birth, regardless of tendon damage. In tenotomy, Scx-positive cells and αSMA-positive cells were accumulated in the stumps of the torn tendon 3 days after surgery; however, only αSMA-positive cells were accumulated, and no Scx-positive cells were found in the defect region.

On day 14 after tenotomy, Scx-positive cells and non-Scx-positive cells were accumulated in the stumps and defect regions, and no αSMA-positive cells were found. TGF-β, known to be important for tendon development, has been shown to play no role in the proliferation of Scx-positive cells, nor in the activity of αSMA-positive cells between day 3 and day 14 after surgery, but has been reported to be important for the activity of non-Scx-positive cells [52,58]. All Scx-positive cells found in neonatal tendons during regeneration have been reported to be derived from tendon tissue [51]. In mechanical tests, tendons 56 days after the operation were indistinguishable from those in the control group. However, the collagen thickness in tendons 56 or more days after surgery was less mature than that in the control group, indicating that the tendon regeneration was not optimal. As a point to be clarified in the future, when TGF-β was inhibited, the expression levels of Scx, Tnmd, and Mkx, which are markers of tendon formation, on day 14 after surgery did not differ from those in the control group. This issue suggests that identifying markers determining the fate of tenocytes will be a focus of future studies.

(2) Tendon regeneration in adult animals

In neonatal tendons, Scx-positive cells are accumulated in the Achilles tendon immediately after birth, as described above. However, Scx-positive cells are usually not accumulated in tendons of adult animals (6–8 weeks) [11]. In a tendon that underwent complete division (tenotomy), Scx-positive cells were observed in the tendon other than the defect region on day 14 after surgery. In adults, the defect site was also filled with new tissue on day 14 after surgery; however, no Scx-positive cells were found in the tissue. The tissue in the defect region has been reported to heal as fibrous scar consisting of αSMA-positive cells. The Scx-positive cells found here included those not derived from the tendon tissue as well as tendon-derived Scx-positive cells. In mechanical tests, regenerated neonatal tendons on day 56 after surgery performed equivalently with the control group; however, the results for regenerated adult tendons on day 56 after surgery were not comparable to those for the control group. These results indicate that tendon regeneration is not mediated exclusively by the tendon tissue in adults and undergoes the scar-forming healing process, and that the regenerated tissue is not mechanically equivalent to the undamaged tissue. Moreover, cartilage formation was observed in the regeneration of adult tendons. Scx-positive cells and aberrant cartilage formation were localized specifically in the injured area of the tendon; this result suggests the interrelation between these two events. In fact, Scx-positive cells were found within cartilage masses. This indicates that, when injuries occur, tenocytes contribute to ectopic ossification through constitutive activation of the BMP receptor ACVR1 [59,60], and different studies have also reported that Scx+/Sox9+ progenitor cells form cartilages or tendons in early embryonic development [61,62]. From these findings, we speculate that reactivation of ScxGFP after injury may reflect the conversion of the tenocyte phenotype to a progenitor-like one. Furthermore, recent studies have demonstrated the possibility that the tendon transcription factor Mkx inhibits cartilage differentiation of tenocytes [63]. The decreased Mkx expression after tendon injuries in adults suggests that cartilage differentiation occurs at the expense of tendon formation. However, studies on the relationship between mechanical stress and tendon progenitor cells are required to address a contradiction that spontaneous cartilage foci are also formed in Scx-null mice.

**Figure 3:**
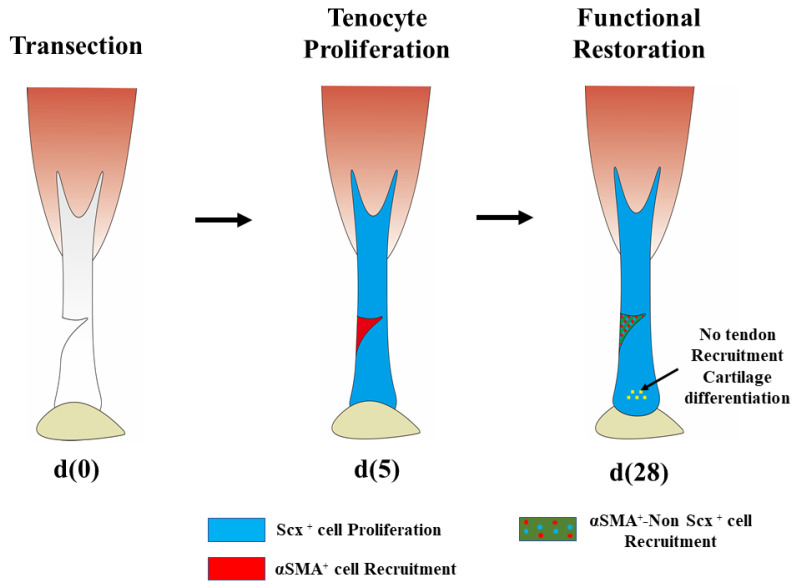
Regeneration of tendons in adult mouse. The time when the mouse Achilles tendon was injured was d(0). And after 5 days, α-SMA positive cells are accumulated in the injured part, and Scx positive cells are accumulated in the tendon tissue other than the injured part: d(5). After another 28 days, α-SMA positive cells and Scx positive cells accumulate in the injured area: d(28). After healing, the tendon tissue shows cartilage-like tissue in the part other than the injured part.

After a partial cut of the tendon tissue (transection) even in adult animals, Sca-1-positive and Scx-negative tendon progenitors localized in the paratenon differentiate into Scx-positive tenocytes, which then migrate to the injured region and play an important role in the adult tendon healing [54]. Specifically, the cells have been suggested to actively produce collagen fibers and repair tendons because type III collagen fibers are replaced by type I collagen fibers. After the damage to the patellar tendon, αSMA-positive cells found in the paratenon have also been reported to become Scx-positive and fill the defect [53,54]. The TGF-β/smad2/3 signals have been reported to induce the Scx expression in progenitor cells after injury and differentiation into tenocytes in adult tendons [64]. However, there also are contradicting reports which show that the excess TGF-β signals induced tenocyte apoptosis [65,66]. The contradicting results warrant future studies focusing on tendon regeneration and TGF-β/smad2/3 signals (Figure 3).

More recent studies have reported that periostin (Postn) contributes to the functional maintenance of tendon stem/progenitor cells (TSPC) and promotes tendon regeneration. The use of periostin-scaffold culture media promotes the recruitment of endogenous TSPCs and collagen fiber organization in a manner close to that before the injury; the tendons regenerated and repaired in this manner had their original mechanical properties and motor function [67]. Further studies are also necessary on periostin and tendon regeneration.

### 3.3. Effects of Mechanical Load on the Growth of Muscle Attachment Sites

The muscle attachment is a special site where the skeletal muscle and the tendon tissue are connected to each other. The tendon and muscle tissues are functionally in a close relationship because collagen fibers of the tendon convey the contraction force of muscle tissue at myotendinous junctions; therefore, microstructures of muscle–tendon junctions have been reported [68]. Furthermore, it is important to maintain the stability of soft tissues, such as muscle attached to bone tissue, in order to maintain the structure of the tissue complex formed by muscle attached to bone [69]. The necessity of muscle load for the development of the musculoskeletal system and for the initiation of formation of bone ridges as cartilage elements in early development suggest the involvement of mechanical load in the attachment formation [70]. Since muscle is required for the growth and maturation of tendons, the tendons may secrete certain factors in response to muscle contraction [27]. There is a debate about whether the contractile force from the muscle affects the growth of this tendon-bone junction. The tendon–bone attachment is mineralized at the final stage of tendon attachment formation. The removal of the muscle load resulted in severe mineralization defects, such as overall volume reduction, morphological changes, and changes in mineral crystal characteristics in the head of the humerus [71]. Future studies may reveal molecules that detect and transmit the mechanical load and induce bone ridge growth and tendon attachment formation. Candidate molecules include growth factors, hedgehog family members, matrix metalloproteinases, angiogenic factors, and other molecules that are involved in mechanical transmission pathways and the formation of tendon–bone attachments.

The tendon and muscle tissues function in a close relationship because collagen fibers of the tendon convey the contraction force of muscle tissue at myotendinous junctions; thus, microstructures of muscle–tendon junctions have been reported [68]. Therefore, TGF-β plays an important role as an inducer of tenogenic differentiation. Meanwhile, TGF-β has the opposite effect of inhibiting collagen fiber maturation, and thus a two-step protocol seemed necessary to achieve effective tendon differentiation [72]. TGF-β has also been reported to effectively promote the formation of tendons and ligaments through interaction with Sox9, a transcription factor of cartilage formation [73]. Furthermore, Scx expressed through BMP4 signals has been known to be involved in the formation of fibrocartilage insertion in joints [74]. These reports have revealed that Sox9- and Scx-positive progenitor cells form fibrocartilage insertion under the control of TGF-β and BMP signals [69]. However, the molecular pathways involved in the formation of tendon attachments have not been fully understood. Since not all bone ridges were lost in BMP4-deficient limbs, other BMPs may be involved in regulating different steps of attachment formation. Moreover, other molecules thought to be involved in tendon-bone attachments include fibroblast growth factor (FGF), which is involved in skeletal morphogenesis and tendon formation.

## 4. Conclusions

Various regulatory mechanisms have been known to control the muscle development process to maturation [75,76,77,78]. Even after birth, the muscle fiber types are switched before and after weaning in mammals, and the muscles then continue to mature until they are matured enough to be responsible for chewing function [79,80,81]. Furthermore, since it is known that regenerated muscles are formed inside the tissue immediately after the necrosis of muscle, the plasticity of the muscle has been investigated from various viewpoints [82,83,84,85]. Recent studies have also revealed that myotendinous junctions, connecting tendon tissues, and bones develop and regenerate as tissue complexes, particularly in muscle attachments, such as the temporomandibular region [86]. Musculoskeletal disorders in elderly people are attributable to the destruction of the muscle–bone connections as well as age-related changes in muscles, and further studies in this area are required.
